# Staphylorrhapy in the Case of Congenital Fissure of the Hard and Soft Palate

**Published:** 1868-05

**Authors:** R. P. Howard, John Bell

**Affiliations:** Professor of Medicine, McGill College.; House Apothecary, Montreal General Hospital.


### ARTICLE IV.

### *Staphylorrhapy in a case of Congenital Fissure of the Hard and Soft Palate.*

By R. P. Howard, M.D., L.R.C.S.E., etc., Professor of Medicine, McGill College.

Reported by John Bell, M.A., M.D., House Apothecary, Montreal General Hospital.

The success of the following operation is due to the untiring perseverance of the surgeon in performing it and the admirable fortitude of the patient James Rowen, who submitted to it. He is a young man from the country, nineteen years of age, healthy and robust.

He was admitted into the wards of the Montreal General Hospital on the 14th of March, 1867, and allowed to remain for several days before the operation was performed, to accustom him somewhat to the hospital air, and by digital manipulation to render the mouth and pharnyx less irritable when touched.

The cleft was quite symmetrical. It commenced within half an inch of the incisor teeth and extended back through the hard and soft palates dividing the uvula longitudinally to its very end. The width of the fissure was about a third of the space between the molar teeth.

On Friday, 22nd March, Dr. Howard performed the operation, the patient sitting and without chloroform. The operation consisted in dividing the *levator* and *tensor palati* muscles midway between the hamular process and the edge of the cleft, then paring the edges of the cleft in the soft palate and bringing them together with fine silk sutures of which six were used.

Passing these sutures through the edges of the cleft formed the most difficult part of the operation. On one side they were introduced by means of short curved needles fixed in a *porte-aiguille*; after being loosened from this instrument the needles were drawn through by means of a pair of long forceps. On the other side of the cleft some of the sutures were introduced by means of fish-hook needles whose eyes were near the blunt end, and others by means of a needle curved like a fish hook near its point, close to which also was its eye, and furnished with a long handle, (originally used for closing a vesico-vaginal fistula.)

By Monday the parts were somewhat inflamed and the sutures covered with lymphy pus. On Wednesday one suture was removed to relieve the tension of the parts, on Thursday two more were removed, on Friday a week from the date of the operation another, and on Saturday the last suture was taken out, leaving more than two-thirds of the part which had been pared, or about one-half of the cleft, firmly united. The inflammatory blush had now almost entirely gone and the parts resumed a very natural color and appearance. The uvula and portion of soft palate portion closed brought together by four stitches united, that by the remaining two, next the hard palate, did not unite either from its extreme tension or from not being brought quite into contact.

For nine days after the operation the patient was not allowed to speak and his diet consisted entirely of fluids. The fissure in the soft palate, was thus closed leaving an oval opening through the hard still to be filled up. On the 4th of April the patient was sent home to the country to allow the tissue to become thoroughly organized and strengthened before any attempt would be made to close the remainder of the fissure.

In a fortnight after his discharge he returned to the Hospital, the object for which he was sent home being now well accomplished. In articulation there was little or no improvement, and the voice had still a sniffling nasal character. April 20th ; To-day, Dr. Howard completed the operation in the following manner. The edges of the opening through the hard palate were pared, an incision was made on each side of the roof of the mouth parallel with the edge of the fissure, and close to the alveolar ridge and the strap of tissue consisting of the mucous membrane, submucous tissue, and periosteum raised completely from the bone by means of a strong laterally curved dull knife. The edges of this strap of tissue which had been previously made raw were now brought together with silk sutures, and cotton wool was inserted into the lateral incisions. Considerable tension was required to bring the edges of the straps of tissue together at the point of union between the soft and hard palate and it was feared that here there would be sloughing.

In this as in the former part of the operation the stitches on one side were introduced by means of a short curved needle fixed in a holder. On the opposite side the difficulty was to get the sutures through from above downwards. This was accomplished by threading a needle of the same kind with the loop end of a doubled thread, passing the needle through the membrane, removing the needle, inserting the upper end of the first suture through the loop and drawing this back through the membrane. All the sutures were introduced before any were tied. A running knot was first tied which was slipped up to the required tightness and on this a common knot was then made.

*Wednesday*, 24*th April.*—Some of the sutures were removed to-day. The two sides have firmly united with the exception of a small space adjoining the soft palate. The cotton still adheres in the wounds. *Friday*—More stitches and the cotton removed. The wounds to be syringed with a weak solution of permanganate of potash. The straps are quite adherent to the bony roof of the mouth. *Monday*—One stitch only remaining. The opening between the soft and hard palate is just large enough to allow a small pea to pass through.

The patient has not spoken since the operation and he has been fed wholly on fluids.

He was now again discharged, Dr. Howard intending at some future period to close up the remaining hole in the palate.

*February*, 24*th*, 1868.—Rowan to-day presented himself at the Hospital for inspection. The aperture left between the portions closed at the two operations is completely filled up, a firm whitish cicatrix, somewhat in the form of a cross, marking the place where it had been. Two raphe-like cicatrices remain in the sites of the lateral incisions, but so nicely have the edges of the cleft coalesced that the line of union can scarcely be pointed out. In articulating, his words have still a very marked nasal sound, to remove which, months, or years even, of vocal gymnastics may be required.

Montreal General Hospital, February 29th, 1868.—*Canada Med. Journal.*

